# The first reptilian circovirus identified infects gut and liver tissues of black-headed pythons

**DOI:** 10.1186/s13567-019-0653-z

**Published:** 2019-05-16

**Authors:** Eda Altan, Steven V. Kubiski, Jennifer Burchell, Elizabeth Bicknese, Xutao Deng, Eric Delwart

**Affiliations:** 1 Vitalant Research Institute, San Francisco, CA 94118 USA; 2 Dept. of Laboratory Medicine, University of California, San Francisco, CA 94118 USA; 3 Institute for Conservation Research, San Diego Zoo Global, San Diego, CA 92112 USA; 4 Veterinary Services, San Diego Zoo Global, San Diego, CA 92112 USA

## Abstract

Viral metagenomic analysis of the liver of a black headed python (*Aspidites melanocephalus*) euthanized for a proliferative spinal lesion of unknown etiology yielded the first characterized genome of a reptile-infecting circovirus (black-headed python circovirus or BhPyCV). BhPyCV-specific in situ hybridization (ISH) showed that viral nucleic acids were strongly expressed in the intestinal lining and mucosa and multifocally in the liver. To investigate the presence of this virus in other snakes and its possible pathogenicity, 17 snakes in the python family with spinal disease were screened with ISH yielding a second BhP positive in intestinal tissue, and a Boelen’s python (*Morelia boeleni*) positive in the liver. BhPyCV specific PCR was used to screen available frozen tissues from 13 of these pythons, four additional deceased pythons with and without spinal disease, and fecal samples from 37 live snakes of multiple species with unknown disease status. PCR detected multiple positive tissues in both of the ISH positive BhP and in the feces of another two live BhP and two live annulated tree boas (*Corallus annulatus*). Preliminary analysis indicates this circovirus can infect BhPs where it was found in 4/5 BhPs tested (2/2 with spinal disease, 2/3 live with unknown status), Boelen’s python (1/2 with spinal disease), and annulated tree boa (2/6 live with unknown status) but was not detected in other python species with the same spinal lesions. This circovirus’ causal or contributory role in spinal disease remains speculative and not well supported by these initial data.

**Electronic supplementary material:**

The online version of this article (10.1186/s13567-019-0653-z) contains supplementary material, which is available to authorized users.

## Introduction

Circoviruses have some of the smallest viral genomes ranging in size from 1.7 to 2.1 kb of circular ssDNA encoding two major proteins, the replication-associated protein (Rep) involved in genome replication and the nucleocapsid (Cap) [1]. Most notorious for causing diseases of birds and swine, circovirus infections have also been documented in other species of mammals, and more recently in amphibians and fish [2–5]. Endogenized sections of circovirus genomes have also been detected in the germ line of mammals, birds, amphibian, reptiles, hagfish, bony fish, and in seven species of snakes in the *Viperidae* family and one member of the *Pythonidae* family (Burmese python or *Python bivittatus*) [6, 7]. The current and past host range of circoviruses therefore appears to include a wide range of vertebrates. A sister clade in the *Circoviridae* family named cyclovirus, whose complementary strand is packaged into virions, has been reported in insects and feces from different vertebrates [1]. Endogenized cyclovirus genomes have so far been detected only in the genome of one insect species (elongate twig ant or *Pseudomyrmex gracilis*) indicating that their tropism may be restricted to invertebrates [8].

In general, circoviruses cause diseases of the immune system, with lymphoid necrosis and depletion in primary or secondary lymphoid tissues linked with immunosuppression [9, 10]. Circovirus pathogenicity is also linked to opportunistic secondary infections and has been studied most extensively in pigs infected with porcine circovirus type 2 (PCV2) [11] and in birds infected with Psittacine beak and feather disease virus (BFDV) [12]. In swine, a number of factors including co-infections, viral load and genotype, management conditions, and serostatus have been shown to influence the outcome of circovirus infections [13–15]. Other circoviruses with described disease associations include canine circovirus [16–18], mink circovirus and other avian circoviruses [12, 19, 20].

So called “spinal osteopathy” is an umbrella term used to describe a well-recognized problem in captive reptiles that present with proliferative and degenerative osteochondral lesions in the vertebrae. In some cases, the characteristic lesions can be clearly associated with previous trauma or infections, while others have no clear etiology leaving metabolic, toxic, or nutritional etiologies as additional considerations [21, 22]. In the latter cases, lesions have been likened to osteitis deformans, or Paget’s Disease, of humans. In addition to similar pathologic findings seen in spinal osteopathy in reptiles, Paget’s Disease has no clear etiology, with genetic and viral origins considered possibilities [23, 24]. For both diseases, the development of lesions is likely complicated and multifactorial.

Here we used available tissues from a black-headed python (BhP) that was euthanized with severe spinal osteopathy of unknown etiology and characterize a novel circovirus genome named Black-headed python circovirus 1 strain SDZ9/2017/USA (BhPyCV-1). We then developed specific PCR and in situ hybridization probes to screen additional snakes in the *Pythonidae* and other families with spinal disease and fecal samples from live snakes with unknown disease status.

## Materials and methods

### Case material

Liver collected at post-mortem exam from a 3-year old male BhP euthanized for severe, progressive spinal disease was frozen at −70 and used for viral metagenomics. Archived formalin-fixed, paraffin-embedded sections of GI tract and liver from 17 similarly affected pythons were screened by in situ hybridization (ISH) for the presence of BhPyCV-1 RNA. Archived, frozen tissues from 13 of those snakes, four additional pythons, and prospectively collected fecal samples from a total of 37 additional live snakes of unknown disease status from various species were also used for PCR. All snakes were from the San Diego zoo.

#### Viral discovery

Liver tissue from the index BhP case was homogenized with a hand-held rotor in approximately 10× volume of Phosphate-buffered saline and rapidly frozen and thawed on dry ice 5 times. After centrifugation for 10 min in a table-top microfuge (15 000 ×* g*), supernatant (400 mL) was collected and filtered through a 0.45 mm filter (Millipore). The filtrates were treated with a mixture of DNases (Turbo DNase [Ambion], Baseline-ZERO [Epicentre], benzonase [Novagen]) and RNase (Fermentas) at 37 °C for 90 min to enrich for viral capsid-protected nucleic acids [25]. Nucleic acids were then extracted using magnetic beads of the MagMAX Viral RNA Isolation kit (Ambion) according to the manufacturer’s instructions. Random RT-PCR followed by Nextera™ XT Sample Preparation Kit (Illumina) was used to generate a library for Illumina MiSeq (2 × 250 bases) with dual barcoding as previously described [26]. An in-house analysis pipeline was used to analyze sequence data. Paired-end reads of 250 bp generated by MiSeq were debarcoded using vendor software from Illumina. Adaptor and primer sequences are trimmed using the default parameters of VecScreen [27]. We developed a strategy that integrates the sequential use of de Bruijn graph (DBG) and overlap-layout-consensus assemblers (OLC) with a novel partitioned sub-assembly approach called ENSEMBLE [28]. Sequence reads were first analyzed using BLASTx (version 2.2.7) for translated protein sequence similarity to all viral protein sequences in GenBank’s virus RefSeq database plus protein sequences taxonomically annotated as viral in GenBank’s non-redundant database using E-value cutoff of 0.001. To remove background due to sequence misclassification these initial viral hits were then compared to all protein sequences in NR using the program DIAMOND (version 0.9.6) and retained only when the top hit was to a sequence annotated as viral. To align reads and contigs to reference viral genomes from GenBank and generate complete genome sequences the Geneious R10 program was used.

The amino acid (aa) pairwise alignments of Rep and Cap protein were performed by the Genious using the in-built MUSCLE algorithm. The amino acid phylogenetic trees were constructed using the Maximum likelihood method with two substitution models: Le Gascuel 2008 model based model with gamma distributed (G+) for Rep and Cap in MEGA software version 6 [29, 30].

#### PCR to confirm long intergenic region

After generation of a complete genome, the repeated region was shown in the long intergenic region. To confirming that region, we designed nested PCR primers as follows: for black-headed python circovirus, primers BhPyCV-817F1 and BhPyCV-277R1 were used for the first round of PCR, and primers BhPyCV-1607F2 and BhPyCV-147R2 for the second round of PCR (Table 1). The PCR conditions in these two assays were 95 °C for 5 min, 35 cycles 95 °C for 30 s, 58 °C (for the first or second round) for 30 s, and 72 °C for 1 min, a final extension at 72 °C for 10 min, resulting in an expected amplicon of 649 bp for BhPyCV long intergenic region.

**Table 1:** **PCR primers used in this study**

Primer ID	Sequence (5′ to 3′)	Size	Target
BhPyCV-817F1	ATAAGGGTGGCACGGTTGAG	1 round 1571 bp	long intergenic region
BhPyCV-277R1	ACAAACCCCTGGATATGCGG		
BhPyCV-1607F2	CAAAGTCCAGTACCGGTCCC	2 round 649 bp	
BhPyCV-147R2	AACACCATCTCTTGCTCGGG		
BhPyCV-642F	TGTTATATGGGCCCCCTGGA	1 round 752 bp	rep gene
BhPyCV-1393R1	ACTTCAGACCACGTCCGAAC		
BhPyCV-874R2	CTCAACCGTGCCACCCTTAT	2 round 233 bp	

#### In situ RNA hybridization

RNA in situ hybridization was performed using the RNAscope^®^ 2.5 HD Red Chromogenic Reagent Kit according to the manufacturer’s instructions (Advanced Cell Diagnostics, Newark, CA, USA). Target probes were designed using the custom software as described previously [31], based on BhPyCV GenBank accession number MH368042. 5 μm sections of formalin fixed, paraffin embedded (FFPE) tissue were mounted on AutoFrost^®^ charged adhesion slides (Cancer Diagnostics, Inc, Durham, NC, USA), baked at 60 °C in a dry oven, and deparaffinized. The sections were treated with an endogenous peroxidase blocker for 10 min at room temperature before boiling in a target retrieval solution for 15 min. Protease plus was then applied for 30 min at 40 °C. Target probes were hybridized for 2 h at 40 °C, followed by a series of signal amplification and washing steps. Hybridization signals were detected by chromogenic reactions using Fast Red. Slides were counterstained in 50% hematoxylin for 2 min and decolorized with 0.2% ammonium hydroxide. After rinsing in deionized water, they were dried in a 60 °C oven, dipped in xylene, and cover-slipped using xylene based SHUR/Mount medium (Triangle Biomedical Sciences, Durham, NC, USA). RNA staining signal was identified as red punctate dots. Negative control background staining was evaluated using a probe specific to a bacterial DapB gene.

#### Rep gene polymerase chain reaction assays (PCR)

DNA was extracted from available, archived tissues belonging to python species that had been frozen at −70 °C, and from prospectively collected fecal samples from multiple species. A total 67 tissues or fecal samples were tested representing 53 snakes and 18 different species. The tissue extraction was performed using the Qiagen Dneasy Blood and Tissue Kit (Qiagen, Valencia, CA, USA) was utilized following the manufacturer’s tissue extraction protocol. The sample DNA was eluted at 100 µL. DNA concentration and purity was assessed utilizing the Biotek Synergy Take3 plate system (Biotek, Winooski, VT, USA). The DNA was stored at 4 °C prior to use and stored long-term at −80 °C. Two primer sets designed in house utilizing the Geneious software v10.2.5 were used independently or as a semi-nested PCR reaction to screen frozen tissues for BhPyCV. The first set included forward primer sequence BhPyCV_642F and reverse primer BhPyCV_1393R1 targeting a ~752 bp fragment of the rep gene. The second set consisted of forward primer BhPyCV_642F and reverse primer BhPyCV_874R2 targeting a ~233 bp of the same region (Table 1). PCR assays were performed using the Eppendorf Mastercycler Pro S thermocycling system (Eppendorf, Hauppauge, NY, USA). Each 25 µL reaction contained the following reagents; 12.5 µL of 2× My Taq HS Mix (Bioline), 400 nM of each primer (Eton Bioscience, San Diego, CA, USA). 2 µL of the tissue extracted DNA was added to each reaction. Each sample was run with a known positive control, negative extraction control, and PCR negative control. The first PCR round had the following reaction parameters: 95 °C for 5 min, [95 °C for 30 s, 53.5 °C for 30 s, 72 °C for 90 s] × 40 cycles, 72 °C for 10 min, and held at 4 °C. The second round PCR was identical except that the annealing temperature was at 53.7 °C.  Bands of the expected size were identified using a 1.8% agarose gel stained with ethidium bromide,  gel extracted using the EMD Millipore Ultrafree™-DA (Millipore, Billerica, MA, USA), and Sanger sequenced.

## Results

### Genome characterization

**Figure 1:**
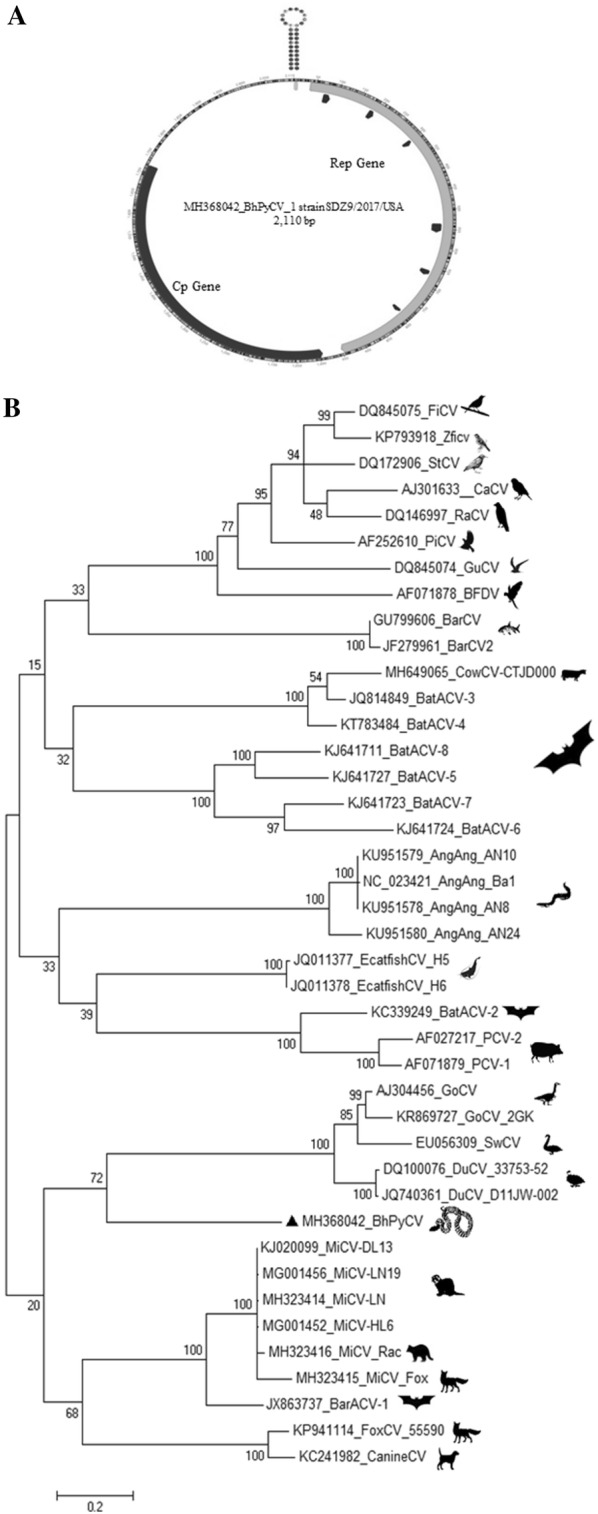
**Characteristics of BhPyCV genome. A** Open reading frames and stem-loop origin of replication of BhPyCV genome. Conserved domains in Rep protein are highlighted. **B** Phylogenetic analysis of circovirus Rep proteins using Maximum Likelihood.

The liver of a BhP with spinal cord defect was processed for viral metagenomics. Viral like particle (VLP)-associated nucleic acids were enriched, randomly amplified and processed for sequencing using the Illumina MiSeq platform (Materials and methods). Nearly 350,000 reads (250 bases long) were generated. The raw sequence data is available at NCBI’s Short Reads Archive under GenBank accession number SRR7982872. Following BLASTx against all known viral protein sequences available in GenBank, a total of 817 reads yielded matches to different circovirus proteins with E score ranging from 1.18e^−67^ to 5.6e^−4^ including a 1527 bases contigs. No other viral contigs nor credible matches to other viral proteins were identified. The contigs were used to design outward-facing PCR primers which were used to fill the gap of the circular DNA genomes by PCR and Sanger sequencing resulting in a 2110 bases genome. Typical of circoviruses, an origin of replication consisting of a 9 base-pair stem-loop topped by unpaired canonical TAGTATTAC sequence was flanked by a Rep protein open reading frame (ORF) encoded on the same strand and a Cap protein ORF encoded on the complementary strand (Figure 1A). The expected Rep rolling circle replication and SF3 helicase motifs were all detected (Figure 1A, Additional file 1) [1]. As typical of circovirus nucleocapsid sequences, its amino acid terminus was rich in basic amino acids (MYRRHFRRRFHHKRHTHFRRYKRHFHHKKR).

An unusually large untranslated region (UTR) of 508 bases was found between the start codon of the Rep and Cap genes (Figure 1A). The length of this UTR was confirmed by nested PCR using PCR primers located within the Rep and Cap genes and Sanger sequencing. The Rep protein showed closest, yet distant, similarity with the Rep of mink circovirus (GB MG001456) with 51% identity (over 92% protein) followed by goose circovirus (GB KR869727) with 50% identity (over 93% protein) (Additional file 2). Sequence alignments showed that the Cap protein of the BhPyCV showed closest (although distant) similarity to a circovirus (BtCV LJR22) of a Least horseshoe bat (*Rhinolophus pusillus*) from China (GB MF278661) with 26% identity (over 81% of protein) (Additional file 2). That bat circovirus genome also showed an unusually long UTR of 450 bases while other circovirus UTR have length ranging from ~70 to 240 bases. The next best capsid protein alignment was with PCV2 with 21% identity over 78% of protein followed by capsids from bird circoviruses (Additional file 2). Phylogenetic analysis of the python circovirus Rep is shown showing its deep branch to other circovirus Rep with a weakly supported branch (72/100) with water fowl-infecting circoviruses (Figure 1B).

### RNA in situ hybridization

**Figure 2:**
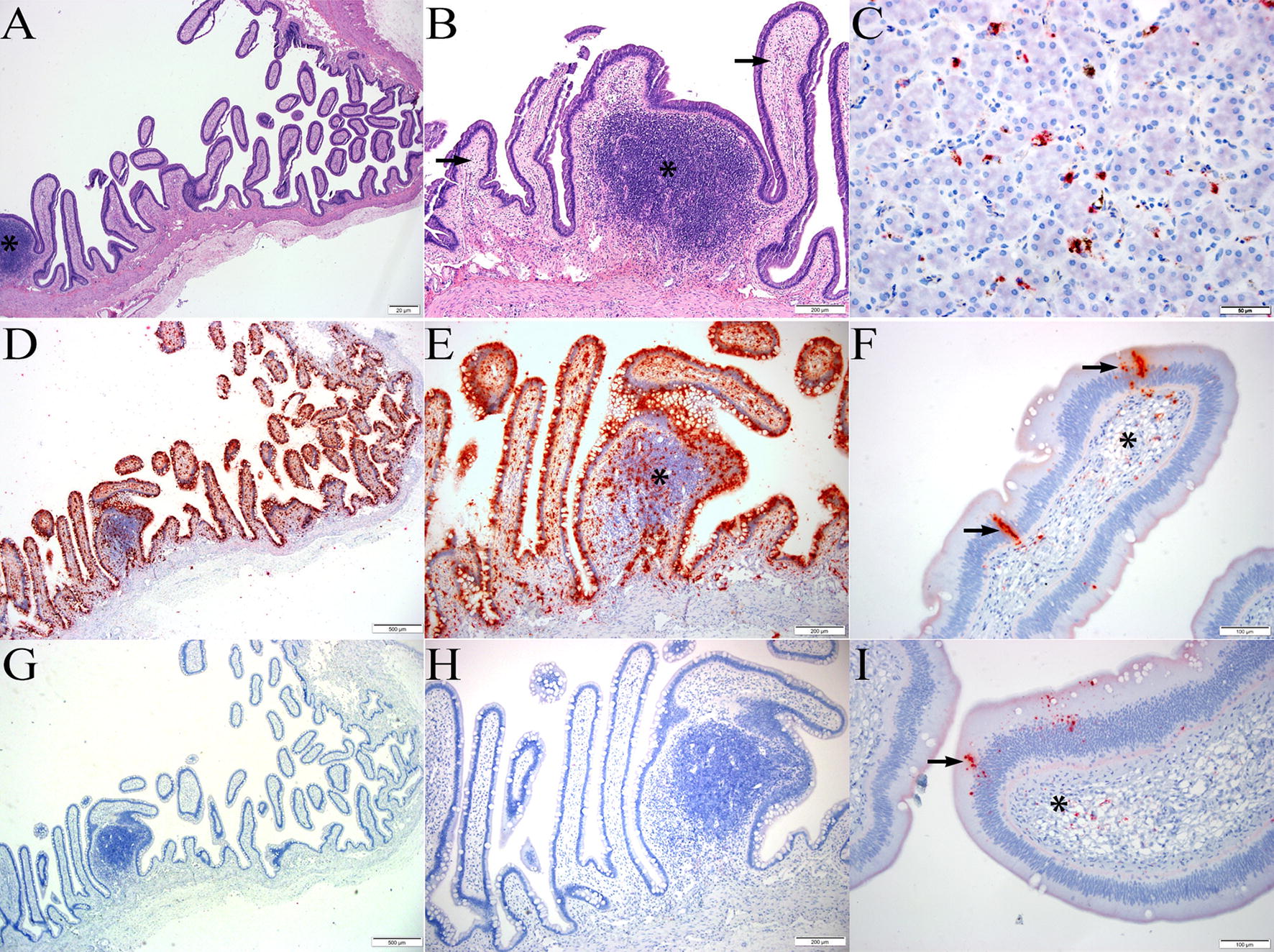
**BhPyCV infection. A**, **B** Longitudinal section of small intestine from index BhP at low (×4, **A**) and higher magnification (×10, **B**) showing a mucosal lymphoid aggregate (asterisk) and villi mildly expanded by edema and lymphocytes (arrows), H&E. **D**, **E** Serial sections from **A**–**B** showing abundant, deep red, punctate to coalescing ISH signal lining the mucosal epithelium, filling intestinal villi, and scattered in lymphoid follicle (asterisk). Hematoxylin counterstain, ×4 (**D**); ×10 (**E**). **G**, **H** Serial histologic sections from **A**–**B**, **D**–**E**. Negative control using bacterial DapB gene probe with no positive ISH signal, hematoxylin counterstain, ×4 (**G**); ×10 (**H**). **C** BhPyCV ISH probe on liver from index BhP showing discrete, punctate red staining within sinusoidal macrophages. Hematoxylin counterstain, ×40. **F**, **I** Medium–high magnification of intestinal villi from second BhP showing similar yet less dense and frequent staining within the epithelium (arrows) and scattered in the lamina propria (asterisk). Hematoxylin counterstain, ×20.

RNA ISH was used to confirm infection of the original snake as well as screen available tissues from additional *Pythonidae* species. In the index BhP (#60415), positive ISH signal was intense throughout the cell lining and mucosa of the small intestine, extending from the superficial submucosa to the tips of villi (Figure 2). Signal was strongest along the epithelial lining with numerous positive cells in the lamina propria which was mildly edematous with some loosely scattered lymphocytes identified on routine staining. Individualized to coalescent signal was seen in submucosal lymphoid follicles and more loosely dispersed in the submucosal stroma. In the liver, positive signal was interpreted as similar bright red and punctate dots within sinusoidal melanomacrophage centers.

An additional 17 snakes in the family *Pythonidae*, all of which have been diagnosed with spinal osteoarthropathy, had both liver and GI tissues screened with ISH. One other BhP showed strong but much less frequent staining in the intestinal mucosa only, and a Boelen’s python had similar staining in the liver (Figure 2). The remainder of pythons had what was considered negative or equivocal staining characterized by rare, single, punctate dots at the basolateral aspect of intestinal epithelium or localized to the nuclear membrane (not shown). No other staining was seen in the liver or intestine of the snakes screened and no similar staining appeared in any of the negative control slides stained with unrelated probes. Negative by ISH were gut and liver tissues from 10 Green tree pythons, 2 carpet pythons, 2 diamond python, and another Boelen’s pythons all exhibiting the spinal defect (Table 2).

**Table 2:** **Deceased snakes tissues with spinal defect screened for BhPyCV with ISH and PCR**

	ISH liver and gut	PCR liver
Species	Animal tested/positive	Animal tested/positive
Black-headed python	2/2^a^	2/2^a^
Ball python	0/0	1/0
Boelen’s python	2/1^b^	1/0^b^
Burmese python	0/0	2/0
Carpet python	2/0	0/0
Diamond python	2/0	0/0
Green tree python	10/0^c^	10/0^c^
Scrub python	0/0	1/0

^a^Same two animals tested by ISH and PCR. Tissues tested/PCR positive: liver (2/2), spleen (2/2), bone (2/2), lung (1/2), kidney (1/2).

^b^Only the ISH negative Boelen’s python tested by PCR.

^c^Same ten snakes tested by ISH and PCR.

### PCR

Archived tissues from python tissues frozen at −70 were screened by hemi-nested PCR using BhPyCV-specific primers. Additionally, DNA from fecal samples collected prospectively to screen for Cryptosporidium infection were opportunistically tested for BhPyCV DNA. A summary of PCR results is presented in Table 2. In total six snakes tested positive, the two ISH positive BhP which died with spinal disease (including the index BhP) from whom multiple tissues including liver, spleen, lung, bone, and spleen were PCR positive (Table 2). Feces from another four live snakes were PCR positive, 2/3 BhP and 2/6 annulated boas with unknown disease status (Table 3). Feces (*n* = 28) from another 10 snake species and tissues (*n* = 4) from another 3 snakes species also with unknown status were also PCR negative. PCR products were Sanger sequenced showing nucleotide identity of 99.5 to 100% with the sequence from the index BhP.

**Table 3:** **Feces from live snakes with unknown diseases status screened for BhPyCV by PCR**

Species	Animal tested	Animal positive
African bush viper	2	0
Angolan python	8	0
Annulated tree boa	6	2
Banded water cobra	1	0
Black-headed python	3	2
Eastern indigo snake	2	0
Eyelash viper	2	0
Green anaconda	2	0
King cobra	1	0
Madagascar tree boa	1	0
Mangshan pitviper	2	0
Rattleless rattlesnake	7	0

## Discussion

We describe here the first circovirus genome replicating in a reptile, the Black-headed Python (*Aspidites melanocephalus*). The detection of an exogenous snake circovirus was anticipated by the report of endogenized circovirus genome remnant in the chromosomes of species in the Pythonidae and Viperidae families [6, 7]. The exogenous circovirus genome described here was not the closest extant relative of the endogenized rep sequences (over an only partially retained Rep encoding region) found integrated in genomes of the family Viperidae (the rattlesnakes *Crotalus horridus* and *Crotalus mitchellii*) and family Pythonidae (the Burmese Python, *Python bivittatus*) (Additional file 3). These results indicate that further exogenous snake circoviruses likely remain to be characterized including lineages more closely related to endogenized circoviral elements [6, 7].

BhPyCV showed typical bidirectional rep and cap ORFs genome architecture with low sequence identity with other circoviruses and an unusually long UTR over the stem loop origin of replication.

The virus was detected by ISH in liver and/or gut of three deceased snakes with severe, non-inflammatory lesions of bone (two BhP and one Boelen’s python). In both BhP cases, DNA extracted from bone was also PCR positive. Feces from another 2/3 BhP and from 2/5 annulated boas of unknown disease status were also PCR positive indicating that enteric replication likely also occurs in both species. However, a number of other pythonidae snakes with the same syndrome were PCR negative in liver and by ISH in liver and GI tract, making the correlation between circoviral infection and spinal disease speculative and currently unsupported by this preliminary screening.

While spinal osteopathy has been linked to infections [21], our cases were considered less likely to be infectious given the lack of an inflammatory response and the dramatic nature to the lesions. Metabolic, toxic or nutritional etiologies remain under strong consideration. Nevertheless, the discovery of a novel virus in this population warrants additional investigation. Not all infections, particularly with viruses, cause significant inflammatory reactions. Many viruses are capable of replicating in tissue and employing mechanisms to evade surveillance and removal by the immune system, including circoviruses [32]. Tissue inflammatory mediators in the absence of a cellular response could also play a role in the development of spinal osteopathy. While numerous species in this collection are affected, often the affected tissues (vertebral bone) are unavailable for screening by ISH and PCR. Routinely collected samples at post-mortem do not typically include bone, and traditional decalcification in hydrochloric acid precludes the ability to run RNA ISH. Therefore, prospective collection of target tissues including affected and unaffected bone as well as optimization of the ISH on decalcified bone is warranted.

Lastly, a previous inflammatory process seeding the vertebral vessels and affecting the bones and joints of the vertebrae cannot be ruled out. Veterinary pathologists with exotic experience consider reptilian bone a dynamic tissue with the propensity to react to different injuries in an exuberate fashion. Therefore prior bone and joint damage could lead to instability and subsequent, ongoing osteochondral proliferation. There is evidence that once infected, circoviruses persist in tissue [33–35]. Stress, co-infections, and other factors could be associated with recurrent bouts of virus replication, compounding inflammatory tissue damage over time. This emphasizes the importance of prospective screening of bone. With more target tissue to evaluate, case–control studies should yield stronger conclusions about the association between circovirus and spinal osteopathy.

Regardless of the relationship of circovirus to spinal osteopathy in snakes, its role in other diseases, particularly as a co-factor in infectious/inflammatory diseases, should also be considered. It is not uncommon for snakes to have inflammatory diseases of the GI, respiratory and reproductive tracts, often with either commensal or opportunistic organisms detected via culture. The possibility of a predisposing circovirus infection can therefore also be considered.

## Additional files

**Additional file 1.** **Conserved motifs of Rep detected in Black headed python circovirus 1 based on [** 1 **].**

**Additional file 2.** **Pairwise amino acid sequence identity values between the Rep and Cap of BhPyCV and those of the most closely related circoviruses.**

**Additional file 3.** **Alignment, protein distances, and phylogenetic analysis of partial Rep of BhPyCV, endogenized snake circoviruses (Serpentes taxa) and exogeneous circoviruses.**

## Abbreviations

Rep: replication-associated protein
Cap: nucleocapsid
PCV2: porcine circovirus type 2
BFDV: Psittacine beak and feather disease virus
BhP: black-headed python
BhPyCV: black-headed python circovirus
ISH: in situ hybridization
DBG: de Bruijn graph
OLC: overlap-layout-consensus assemblers
aa: amino acid
G+: gamma distributed
FFPE: formalin fixed, paraffin embedded
PCR: polymerase chain reaction assays
VLP: viral like particle
ORF: open reading frame
UTR: untranslated region
GB: GenBank
