# Supplementary material for: The first reptilian circovirus identified infects gut and liver tissues of black-headed pythons
Source: Vet Res. 2019 May 16;50:35. doi: 10.1186/s13567-019-0653-z (PMC6524214; doi:10.1186/s13567-019-0653-z)
Supplement: Supplementary file 1 — Additional file 1. Conserved motifs of Rep detected in Black headed python circovirus 1 based on [ 1 ]. [file 13567_2019_653_MOESM1_ESM.docx]

**Additional file 1** **Motifs of Rep detected in Black headed python circovirus 1 based on [1].**

| **Name of region** |  | **Sequence of Circoviridae family** | **Sequence of Black headed phyton circovirus 1** |
| --- | --- | --- | --- |
| **RCR motif** | RCR motif I | FT(L/I)NN | CFTINN |
|  | RCR motif II | PHLQG | PHIQG |
|  | RCR motif III | YC(S/x)K | YCSK |
| **SH3 helicase motif** | Walker A | G(P/x)(P/x) GxGK(S/t) | GPPGCGKS |
|  | Walker B | uuDDF | VLDDF |
|  | Walker C | uTSN | ITSN |
