# Supplementary material for: The first reptilian circovirus identified infects gut and liver tissues of black-headed pythons
Source: Vet Res. 2019 May 16;50:35. doi: 10.1186/s13567-019-0653-z (PMC6524214; doi:10.1186/s13567-019-0653-z)
Supplement: Supplementary file 2 — Additional file 2. Pairwise amino acid sequence identity values between the Rep and Cap of BhPyCV and those of the most closely related circoviruses. [file 13567_2019_653_MOESM2_ESM.docx]

**Additional file 2 Pairwise amino acid sequence identity values between the study sequence (MH368042_BhPyCV 1) and the most closely related circoviruses.**

|  | Black headed python circovirus BhPyCV1 MH368042  % identity | |
| --- | --- | --- |
|  | Rep protein | Cap protein |
| MG001456_MiCV-LN19 | 51 | 17 |
| KR869727_GoCV_2GK | 50 | 15 |
| DQ100076_DuCV | 49 | 17 |
| EU056309_SwCV | 48 | 17 |
| JX863737_BarACV-1 | 48 | 16 |
| DQ845075_FiCV | 48 | 20 |
| JQ814849_BatACV-3 | 47 | 20 |
| DQ172906_StCV | 47 | 17 |
| KP793918_Zficv | 47 | 19 |
| MH649065_CowCV | 46 | 17 |
| AJ301633 _CaCV | 46 | 18 |
| KT783484_BatACV-4 | 45 | 19 |
| AF252610_PiCV | 45 | 20 |
| AF071878_BFDV | 44 | 18 |
| DQ146997_RaCV | 44 | 19 |
| MF278661_BtCV-LJ22 | 43 | 26 |
| AF071879_PCV-1 | 43 | 22 |
| KJ641724_BatACV-6 | 43 | 16 |
| GU799606_BarCV | 43 | 18 |
| AF027217 PCV-2 | 42 | 21 |
| JQ011377_EcatfishCV | 42 | 19 |
| KC339249_BatACV-2 | 41 | 21 |
| DQ845074_GuCV | 41 | 18 |
| NC_034210_Bat_Myotis | 41 | 26 |
| KU951578_AngAng_AN8 | 41 | 8 |
