# Supplementary figures and images for: The first reptilian circovirus identified infects gut and liver tissues of black-headed pythons
Source: Vet Res. 2019 May 16;50:35. doi: 10.1186/s13567-019-0653-z (PMC6524214; doi:10.1186/s13567-019-0653-z)

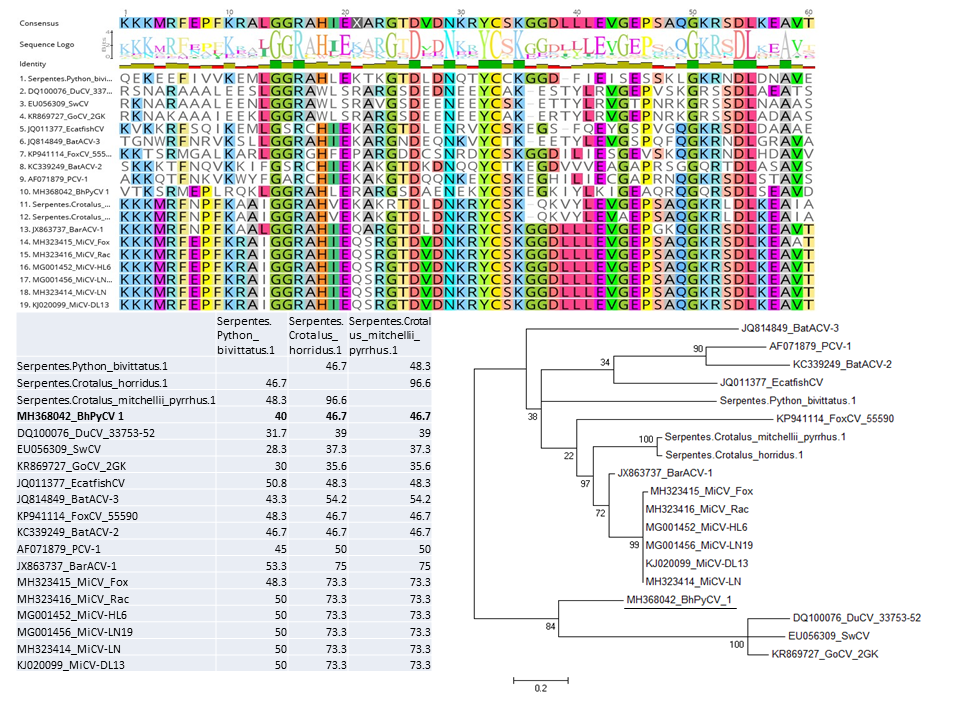

Supplement: Supplementary file 3 — Additional file 3. Alignment, protein distances, and phylogenetic analysis of partial Rep of BhPyCV, endogenized snake circoviruses (Serpentes taxa) and exogeneous circoviruses. [file 13567_2019_653_MOESM3_ESM.tif]
